# Layer-by-Layer Engineered Flexible Functional Film Fabrication with Spreadability Control in Roll-to-Roll Manufacturing

**DOI:** 10.3390/polym15112478

**Published:** 2023-05-27

**Authors:** Hojin Jeon, Jaehyun Noh, Minho Jo, Changbeom Joo, Jeongdai Jo, Changwoo Lee

**Affiliations:** 1 Department of Mechanical Design and Production Engineering, Konkuk University, 120 Neungdong-ro, Gwangjin-gu, Seoul 05029, Republic of Korea; tom2431@konkuk.ac.kr (H.J.); zenty616@konkuk.ac.kr (J.N.); als8080@konkuk.ac.kr (M.J.); 2 Department of Mechanical Engineering, Stevens Institute of Technology, 1 Castle Point Terrace, Hoboken, NJ 07030, USA; cjoo@stevens.edu; 3 Department of Printed Electronics, Korea Institute of Machinery and Materials, 156, Gajeongbuk-ro, Yuseong-gu, Daejeon 34103, Republic of Korea; micro@kimm.re.kr; 4 Department of Mechanical and Aerospace Engineering, Konkuk University, 120 Neungdong-ro, Gwangjin-gu, Seoul 05029, Republic of Korea

**Keywords:** functional film, ink spreadability, layered structures, roll-to-roll, slot-die coating, surface roughness

## Abstract

A roll-to-roll manufacturing system performs printing and coating on webs to mass-produce large-area functional films. The functional film of a multilayered structure is composed of layers with different components for performance improvement. The roll-to-roll system is capable of controlling the geometries of the coating and printing layers using process variables. However, research on geometric control using process variables is limited to single-layer structures only. This study entails the development of a method to proactively control the geometry of the upper coated layer by using the lower-layer coating process variable in the manufacture of a double-coated layer. The correlation between the lower-layer coating process variable and upper coated layer geometry was examined by analyzing the lower-layer surface roughness and spreadability of the upper-layer coating ink. The correlation analysis results demonstrate that tension was the dominant variable in the upper coated layer surface roughness. Additionally, this study found that adjusting the process variable of the lower-layer coating in a double-layered coating process could improve the surface roughness of the upper coating layer by up to 14.9%.

## 1. Introduction

A roll-to-roll manufacturing system is used for the mass production of large-area functional films, and various products can be fabricated based on the process configuration. Polymer-based films transferred through continuously placed rolls in a roll-to-roll manufacturing system are processed into functional films via web conversion processes, including slot-die coating, gravure printing, spray coating, and dip coating [1,2,3,4,5]. Products processed using roll-to-roll printed electronic systems typically include electronic functional devices, such as flexible sensors, solid oxide fuel cells (SOFCs), solar cells, and thin-film transistors (TFTs) [6,7,8,9]. These products are composed of various functional layers and perform a single function through the interaction among the functional layers. The roll-to-roll manufacturing system is highly suitable for the mass production of multifunctional layered products as several functional layers can be multiprinted and coated through continuous processes. Lee et al. manufactured a double-layered SOFC electrolyte using slot-die coating in a roll-to-roll system [10]. A yittria-stabilized zirconia–gadolinium-doped ceria (YSZ–GDC) double-layered electrolyte was proposed as a solution to the exfoliation caused by thermal deformation due to the different thermal expansion coefficients of the existing single electrolyte layer compared to the anode [10]. Cho et al. produced TFTs for a 4-bit code generator using a gravure printing system [11]. TFTs consist of a functional gate electrode, a dielectric, an insulator, and active, drain, and source layers printed continuously through gravure printing [11]. Thue et al. produced polymer solar cells using slot-die coating in a roll-to-roll system [12]. The authors conducted a functional quality analysis of polymer solar cells manufactured by a multi-coating method for stacking active and hole-transporting layers (HTLs) simultaneously and a single coating method for sequential stacking [12].

The functional quality of multilayered electronic functional devices manufactured via a roll-to-roll system is affected by the geometric quality of the coating layer [13]. For example, the thinner the SOFC electrolyte layer, the higher the ion conductivity; thus, it is possible to improve the SOFC performance by controlling the electrolyte layer thickness [14]. Additionally, since the performance of TFTs is dependent on the thickness of the dielectric layer, thickness control is required for their dielectric layer coating [15]. Owing to the relationship between coating layer geometry and device performance, several studies have been conducted on the layer geometry control in the roll-to-roll manufacturing of functional devices [3].

Coating layer geometry is controlled by process variables during the roll-to-roll manufacturing of electrical functional devices. In a roll-to-roll manufacturing system, the controllable factors are classified into rheological parameters, including ink viscosity and surface tension, as well as system parameters, including the ink flow rate, web speed for coating, tension, and coating gap [13,16,17]. An increase in the flow rate of the roll-to-roll slot-die coating system increases the coating layer thickness, which is explained by a model derived from the law of mass conservation, as discussed by Kang et al. [3]. Carvalho et al. developed a viscocapillary model for the minimum wet thickness that ensures a stable coating quality [18]. The viscocapillary model includes the process variable of the coating gap, which represents the distance between the coater nozzle and the web [18]. Since tension can be adjusted relatively freely compared to other process variables, various studies have been conducted to control the geometries of the coated and printed layers. Lee et al. reported that as the tension on the web increased, the surface roughness and thickness of the printing pattern decreased and established a trend originating from the ink’s spreadability by measuring the surface roughness of the bare substrate and the contact angle [13]. Jo et al. recognized that the geometries of TFTs’ conductive and dielectric layers change the printing pattern as per the ink’s spreadability and analyzed the change in pattern thickness and width based on the process variables of web speed and tension. Based on these measurements, a metamodel for the pattern geometry was derived using the experimental planning method, and the tendency to change the thickness and width with the web speed and tension was observed [19].

As mentioned above, the functional coating and printing layers processed using a roll-to-roll manufacturing system typically possess a multilayered structure. However, to date, the research on the change in the coating and printing layer geometries based on process variables has primarily been limited to a single layer [13,17,19]. Few studies have been conducted on the thickness of the coating layer based on a single-layer thickness prediction model for multilayered structures [10]. Moreover, in multilayered coating, unlike in single coating layers wherein the tension is applied to the bare substrate, it is applied to the already dried lower coated layer along with the bare substrate simultaneously during the upper-layer coating. Thus, tension may generate an external force greater than the yield strength of the coating layer, leading to crack defects in the coating layer [20]. Since the crack defects caused by the tension applied to the coating layer are directly related to its performance degradation, using tension for the geometric control of the upper coated layer is not advisable [21]. Therefore, other suitable control methods are required for the geometry of the upper coating layer of a multilayered structure.

In this study, a method was devised to employ the process variables in a lower coating layer to control the geometry of the upper coated layer in a multilayered functional coating by using slot-die coating in a roll-to-roll system. In the fabrication of a double-layered structure, the surface roughness of the upper coated layer was measured based on the tension of the lower coated layer and the process variables of the coating gap to analyze the change in layer geometry. The process conditions to create the double structural–functional layer were determined according to the design of experiment (DOE). A regression analysis confirmed that tension had a greater influence on the upper-layer surface roughness. In addition, the change in the contact angle of the upper-layer coating ink with the lower coated layer was analyzed. The analysis results demonstrated that the correlation between the lower-layer coating process variable and the upper coated layer geometry emerged from the ink’s spreadability. Finally, a performance analysis was conducted based on the lower-layer coating process variable of the filler gradient structure graphene nanoplatelet (GNP)/polymethyl methacrylate (PMMA) nanocomposite, forming the functional layer of a double-layered structure layer [22,23,24].

## 2. Theory

**Figure 1:**
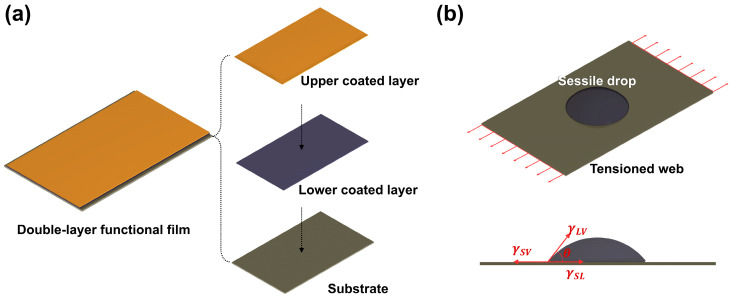
(**a**) Schematic of a double-layered structure’s functional film. (**b**) Contact angle and surface tension of the sessile drop on a tensioned web.

In the fabrication of the double-layered structure functional film of Figure 1a, geometric control of the lower coated layer using the process variables of tension and coating gap was performed through the ink’s spreadability. Among them, the change in the spreadability of the ink by tension applied to the bare substrate is explained by the Young–Dupre equation, which indicates the relationship between the contact angle of the sessile drop, the surface tension of the ink, and the surface energy of the material (Figure 1b) [19].

First, the relationship between the surface tension of the ink and the contact angle is described by Young’s equation, as shown in Equation (1) [25]:

(1) $$\gamma_{SV}=\gamma_{SL}+\gamma_{LV}cos\theta_{Y}$$

where $\theta_{Y}$ and γ denote the Young’s contact angle and the ink’s surface tension, respectively. Subscripts *SV*, *SL*, and *LV* denote the solid–vapor, solid–liquid, and liquid–vapor interfaces, respectively. However, Young’s equation is far from a real phenomenon because it assumes that a surface is chemically uniform and morphologically smooth.

Wenzel defined contact angles reflecting actual surface properties as follows [26]:

(2) $$\cos\;\theta_{W}=r\cos\;\theta_{Y}$$

where $\theta_{W}$ and r represent the Wenzel’s contact angle and the roughening ratio, respectively. r is calculated as the ratio of the actual area (a) to the projection area (A) (*r* = a/A). The Wenzel model shows that the rougher the surface, the more hydrophobic the substrate, whereas the more even the surface, the more hydrophilic the substrate. This indicates that surface roughness affects ink spreadability [27].

Under the assumption that absorption does not occur at the liquid–vapor ($\gamma_{LV}=\gamma_{L}$) and solid–vapor ($\gamma_{SV}=\gamma_{S}$) interfaces, the Young–Dupre equation from Equation (1) can be used to determine the surface energy ($W_{SL}$) [28] as follows:

(3) $$W_{SL}=\gamma_{L}(1+\cos\;\theta_{Y})$$

According to the relations in Equations (1)–(3), a material with a smooth surface has a high surface energy and a small contact angle, i.e., the more even the material surface, the higher the ink spreadability. According to this principle, the spreadability of the ink in a roll-to-roll system is determined by the surface roughness of the bare substrate. Because the surface roughness of the bare substrate decreases with increasing tension to the web, a correlation is established that ink spreadability increases with an increase in the applied tension [19].

## 3. Materials and Methods

Table 1 lists the physical properties of the coating inks used in this study. Although the inks used for the lower- and upper-layer coatings of the GNP/PMMA nanocomposite were composed of the same components, they were characterized by low and high GNP content, respectively, according to the filler content gradient structure for improving the thermal conductivity of the structure [24]. For both inks, GNPs (PD-100; graphene supermarkets, Ronkonkoma, NY, USA) were used as the fillers, (PMMA; Sigma-Aldrich, St. Louis, MO, USA) as the polymer matrix, and N-methyl-2-pyrrolid (NMP; Sigma-Aldrich, St. Louis, MO, USA) as the solvent for the inks. Total amounts of 0.2 g of GNPs and 200 mL of NMP were mixed to prepare the GNP dispersion in each ink. The mixture was ultrasonically stirred for 20 min for mechanical dispersion. Next, 4.5 g of PMMA and 100 mL of NMP were stirred for 2 h using a magnetic stirrer to obtain a PMMA solution. The GNP dispersion and PMMA solution were mixed to obtain a low-GNP-content ink for the lower-layer coating and a high-GNP-content ink for the upper-layer coating, with 4 wt% and 8 wt% GNP/PMMA, respectively. Finally, the ink for the lower and upper layers of the GNP/PMMA nanocomposite coating was fabricated under stirring for 30 min using a magnetic stirrer [29,30,31].

**Figure 2:**
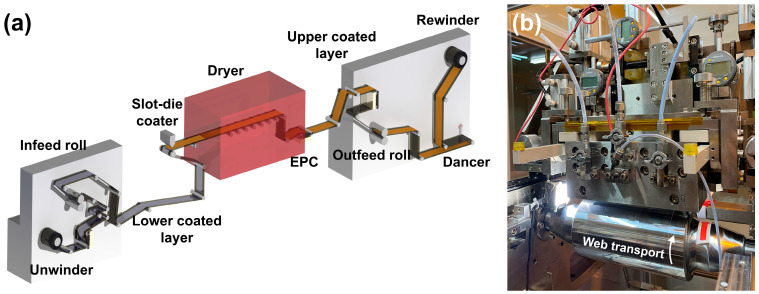
(**a**) Schematic of a double slot-die coating system. (**b**) Slot-die coater.

Figure 2a,b shows a schematic of the slot-die coating in the roll-to-roll system used in this study. The system consists of unwinding, coating, drying, and rewinding sections and is equipped with a dancer and an edge position controller (EPC). This coating system removes tension fluctuation through the dancer’s feedback control and can also control lateral displacement using an EPC, free from tension and register errors [32,33]. The process conditions used in this experiment are listed in Table 2. In slot-die coating, the process conditions must be set based on the ink and web characteristics. The web speed, flow rate, and drying temperature were set considering the length of the dryer in the system to enable the sufficient evaporation of the ink solvent. The dryer of this system is 5 m long and uses hot air and an infrared (IR) heater simultaneously. Considering the drying conditions of the GNP/PMMA nanocomposite, the drying temperature, flow rate, and web speed were set to 65 °C, 3 mL/min, and 1 m/min, respectively. In a roll-to-roll system, the proper operating tension is determined according to the physical properties of the web to be transferred. Hawkins et al. calculated the optimal operating tension of the system for transferring the polymer-based film as 6.67% of the yield stress [34]. Based on the physical properties of the polyethylene terephthalate film (PET film, CH34P; KOLON Inc., Seoul, Republic of Korea) used in this study, the process variable for tension was set to 18.6–55.9 N. Another process variable, the coating gap, was set to the 100–300 μm range permitted by the roll-to-roll slot-die system.

**Figure 3:**
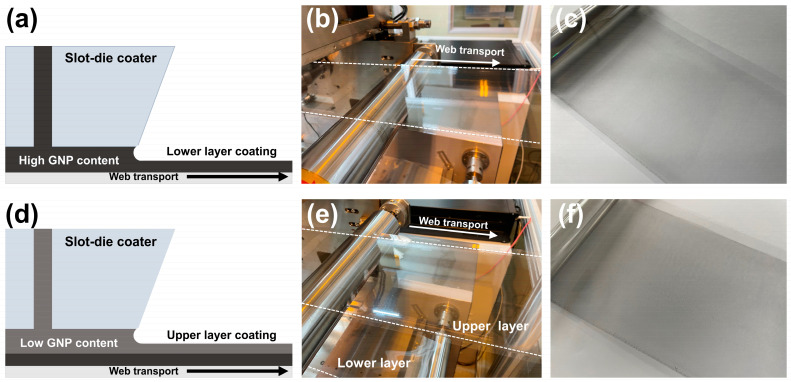
(**a**) Schematic diagram of the lower-layer coating. (**b**) Lower-layer coating process. (**c**) Dried lower-layer coating. (**d**) Schematic diagram of the upper-layer coating. (**e**) Upper-layer coating process. (**f**) Dried double-layered coating.

**Figure 4:**
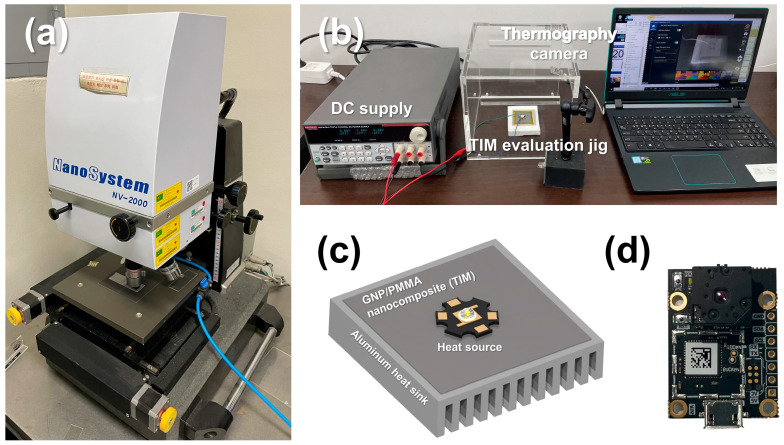
(**a**) Interferometer. (**b**) Evaluation instruments for steady-state temperature analysis. (**c**) Jig for the heat conduction performance of TIMs. (**d**) Thermography camera.

A DOE-based experimental case was established to analyze the relationship between the lower-layer coating process variable and the change in the upper coated layer. Table 3 presents an experimental case prepared by two-factor and three-level full factorial design methods to analyze the change in surface roughness due to the coating gap and tension process variables. Figure 3a is a schematic diagram of the lower-layer coating process using slot-die coating. The ink discharged from the slot-die coater nozzles forms a wet coated layer, and the solvent is dried by the subsequent drying process to form a completed coating layer (Figure 3b,c). Figure 3d is a schematic diagram of the upper-layer coating, and the same coating process was repeated on the upper part of the previously manufactured lower coating layer to form a double-layered coating layer (Figure 3e,f). The upper-layer coating was put under certain process conditions (tension = 18.6 N, coating gap = 200 μm) to check the effect of the surface characteristics of the lower coating layer. The surface roughness of the lower coating layer was measured according to the process variables of each case, and the surface roughness of the upper coating layer stacked on top was also measured using an interferometer (NV-2000; NanoSystem Co., Ltd., Daejeon, Republic of Korea), as shown in Figure 4a.

Additionally, the functional quality of the GNP/PMMA nanocomposite with a double-layered structure manufactured using slot-die coating was analyzed. We used the performance evaluation method of thermal interface materials (TIMs), a practical application of polymer-based thermal nanocomposites, including GNP/PMMA nanocomposites [35,36,37]. TIMs are mainly located between the heat sink and source to prevent the thermal weathering of the integrated circuit and function as thermal conductors. Therefore, the thermal conductivity of the GNP/PMMA nanocomposite was selected as the functional quality, and a steady-state temperature analysis method was applied [24,38]. For the steady-state temperature analysis, a jig consisting of a thermography camera (LEPTON 3.5; Teledyne FLIR, Wilsonville, OR, USA), GNP/PMMA nanocomposite, aluminum heatsink, DC supply (2231A-30-3; Keithley, Solon, OH, USA), and 1 W LED was produced, as shown in Figure 4b–d [39]. The 1 W LED is a heating element that received constant voltage from the DC supply. The GNP/PMMA nanocomposite is the TIM between the aluminum heat sink and the heating element. Therefore, the heat conduction performance of the GNP/PMMA nanocomposite was determined according to the steady-state temperature in the corresponding jig [24].

## 4. Results and Discussion

**Figure 5:**
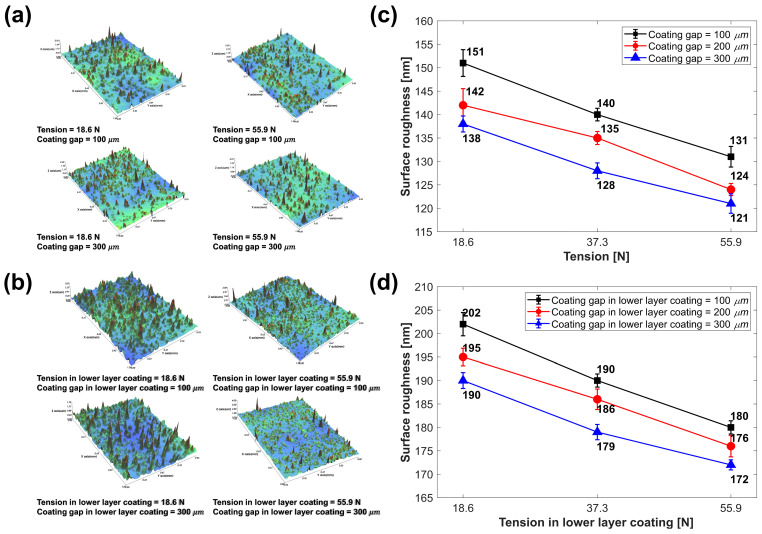
(**a**) Measurement of the lower-layer surface roughness (Cases 1, 3, 9, and 7 clockwise). (**b**) Measurement of the upper-layer surface roughness (Cases 1, 3, 9, and 7 clockwise). (**c**) Changes in the lower coated layer’s surface roughness. (**d**) Changes in the upper-layer surface roughness with the process variables of the lower-layer coating.

Figure 5a,b depicts images of the lower- and upper-layer surface of the GNP/PMMA nanocomposite manufactured in this study. Figure 5c shows the change in the lower coated layer’s surface roughness with the coating gap and the tension in the lower-layer coating. First, as the web tension increased under the constant coating gap condition, the lower-layer surface roughness decreased, and the maximum decrease in the surface roughness was observed (from 151 to 131 nm, a 13.2% reduction for 18.6 to 55.9 N) under the 100 μm coating gap condition. As previously mentioned, this is a trend caused by an increase in ink spreadability due to a decrease in the surface roughness of the bare substrate as the applied tension increases [19].

In addition, as the coating gap increased under all tension conditions, the surface roughness of the lower layer decreased. As shown in Figure 5c, a maximum decrease in the surface roughness (151 to 138 nm, an 8.6% decrease from 100 to 300 μm) was observed under the 18.6 N tension condition. The potential energy of the ink discharged from the coater nozzle is determined by the coating gap. Accordingly, the ink’s potential energy according to the coating gap determines the inertial effect while reaching the substrate surface from the coater, thus determining the ink’s spreadability in reaching the substrate surface. The potential energy of the discharged ink increases with the coating gap, thereby increasing the ink’s spreadability [17].

Figure 5d shows the measurement images for the surface of the upper coated layer of the GNP/PMMA nanocomposite. Although the upper-layer coating process variables were maintained under certain conditions (tension = 18.6 N, coating gap = 200 μm), the coating surface roughness was different in each case, classified according to the lower-layer coating process variables. The changes in the surface roughness of the upper and lower coated layers were the same. As the tension in the lower-layer coating increased, the surface roughness of the upper coated layer decreased by up to 10.9% (Cases 1 and 3). As the coating gap in the lower-layer coating increased, the surface roughness of the upper coated layer decreased by up to 5.9% (Cases 1 and 7). These results demonstrate that the geometric changes in the upper- and lower-layer coating process variables have a close correlation.

**Figure 6:**
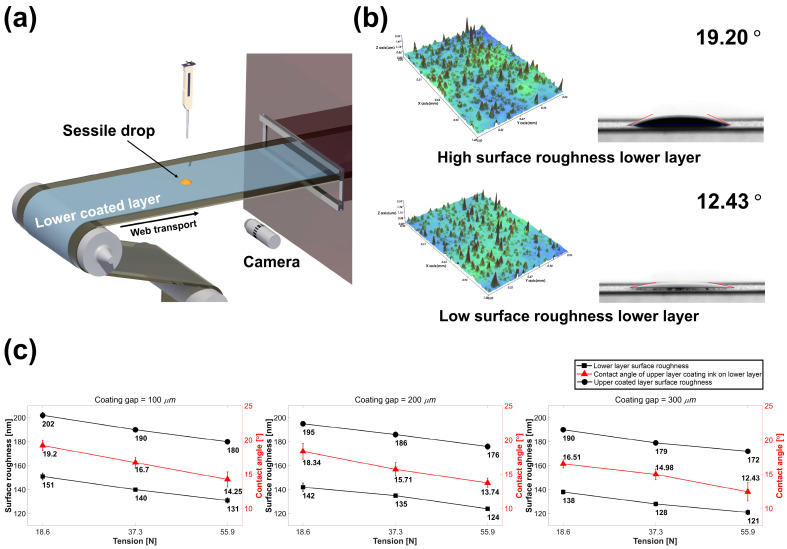
(**a**) Schematic of sessile drop analysis in a roll-to-roll system. (**b**) Contact angle changes with lower-layer surface roughness. (**c**) Measurement of the upper-layer coating ink contact angle on the lower coated layer according to the coating gap and tension in the lower-layer coating.

To analyze the correlation between the changes in the surface roughness of the upper- and lower-layer coating process variables, the contact angle of the upper coated layer ink spread with respect to the lower coated layer was measured through sessile drop analysis. A jig for the sessile drop analysis in the roll-to-roll system was installed (Figure 6a), and the image of the contact angle was taken by a camera (AM7915MZT; AnMo Electronics Co. Ltd., New Taipei City, Taiwan). The lower coated layer manufactured under the process conditions of Cases 1–9 was used as the substrate, and sessile drop analysis was performed using the GNP/PMMA ink for the upper-layer coating. Figure 6b shows the contact angle of the upper-layer coating ink spread for the lower coated layer according to the lower-layer coating conditions. The contact angle of the upper-layer coating ink decreased as the coating gap and tension in the lower-layer coating increased. This implies that the contact angle decreased by 25.8% as the tension increased under a coating gap condition of 100 μm (from 19.20° to 14.25°, Cases 1 and 3) and by 14.0% as the coating gap increased under the tension of 18.6 N (from 19.20° to 16.51°, Cases 1 and 7), i.e., the spreadability of the upper-layer coating ink varied according to the lower-layer coating process variables.

Figure 6c shows the surface roughness measurement results of the lower and upper coated layers of the GNP/PMMA nanocomposite and the change in the upper-layer coating ink contact angle for the lower coated layer according to the lower-layer coating process variables. Each measurement result shows that there is a constant tendency depending on the tension and coating gap in the lower-layer coating. As the tension in the lower-layer coating increased, the lower coated layer surface roughness, upper-layer coating ink contact angle, and upper coated layer surface roughness decreased. Moreover, as the coating gap increased in the lower-layer coating, the lower coated layer surface roughness, upper-layer coating ink contact angle, and upper coated layer surface roughness decreased. As described above, a constant trend occurred in the change in the surface roughness of the lower layer owing to the process variables. As the tension in the lower-layer coating increases, the surface roughness of the bare substrate decreased, and the resulting increase in the ink spread caused a decrease in the surface roughness of the lower layer [19]. In addition, the potential energy of the ink increased as the coating gap increased, which increased the ink’s spreadability and decreased the roughness of the lower layer [17].

The lower coated layer, with different surface roughness values depending on the process variables, acts as a factor in changing the ink’s spreadability in the subsequent upper-layer coating. In the system under examination, the lower coated layer exhibited a higher surface energy as the surface roughness decreased, resulting in an increase in the spreadability of the upper-layer coating ink. The change in the ink’s spreadability of the upper-layer coating also induced a change in its surface roughness. Therefore, the correlation between the lower-layer coating process variables and geometry of the upper coated layer can be explained by the influence of the ink’s spreadability according to the surface roughness acting in a chain.

**Figure 7:**
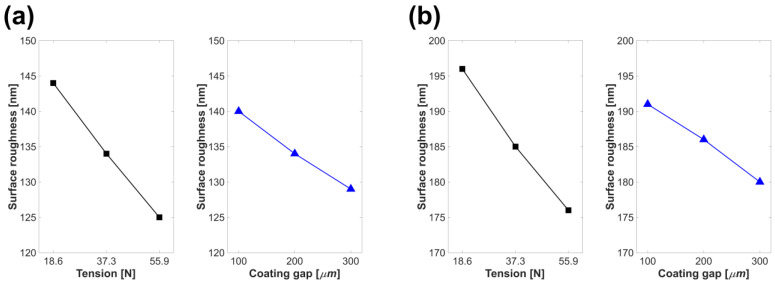
Correlations between the system parameters and coated layer surface roughness: (**a**) lower layer (nm) and (**b**) upper layer (nm).

Based on the measurement results, a regression analysis was conducted with the lower-layer coating tension, coating gap, and lower and upper coated layer surface roughness as factors and reactions [40,41]. The main effect graphs of each factor in Figure 7a,b show the process variables of tension and coating gap in the lower-layer coating and surface roughness of the lower and upper coated layers, respectively. Table 4 and Table 5 present the results of the variance analysis of the lower and upper coated layers, respectively. The F-values indicate the effects of each factor on the response, and the *p*-values indicate the significance of these effects [42]. As a result of the analysis of variance (ANOVA), the F-values for tension of the lower- and upper-layer surface roughness were 62.44 and 154.32, respectively, while those of the coating gap, were 27.75 and 56.60, respectively. The F-values confirmed the significance of these factors, as they exceeded the critical F-value of 6.61 in the 95% confidence interval of the regression model of the lower and upper coated layers. In addition, each ANOVA table shows that the F-value of the tension was larger than that of the coating gap, clearly indicating that tension had a greater effect on surface roughness. Meanwhile, each *p*-value confirmed that the coating gap and tension acted significantly in the 95% confidence interval of the surface roughness of the lower and upper coating layers; however, the interaction effect between the coating gap and tension was not statistically significant.

Equations (4) and (5) are the metamodels for the surface roughness of the lower and upper coated layers, respectively, derived through regression analysis. Herein, *R*, *l*, *u*, *T*, and *h* represent the surface roughness, lower layer, upper layer, tension, and process variable of the coating gap, respectively.

(4) $$R_{l}=168.11-5.789\;T-0.0733\;h+0.00395\;T*h$$

(5) $$R_{u}=219.56-6.228\;T-0.07167\;h+0.00526\;T*h$$

The R-squared values of the metamodel for the lower and upper coated layers were 98.90% and 99.48%, respectively, indicating a high accuracy in predicting the surface roughness [43,44].

**Figure 8:**
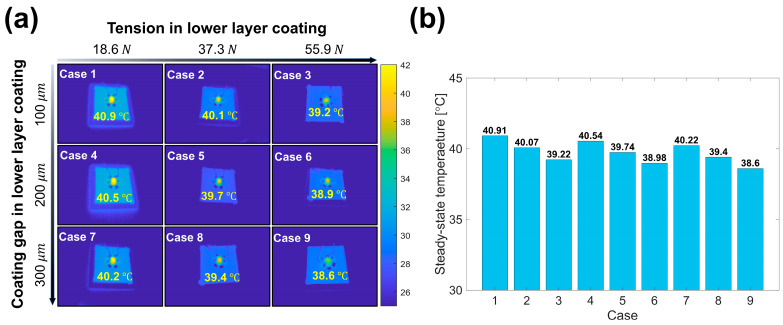
(**a**) Infrared image of the steady-state operating temperature of the experimental cases. (**b**) Steady-state operating temperatures of the experimental cases.

Figure 8a,b shows the thermal image and steady-state temperature according to the lower-layer coating cases as the temperature of the heating element reached a steady-state. According to the measurement results, the steady-state temperature decreased as the GNP/PMMA nanocomposite was manufactured under the conditions of high tension and a coating gap in the lower-layer coating. The steady-state temperature decreased by 4.1% in Case 3 (coating gap = 100 μm, tension = 55.9 N) and by 1.7% in Case 7 (coating gap = 300 μm, tension = 18.6 N) compared to Case 1 (coating gap = 100 μm, tension = 18.6 N). A decrease in the steady-state temperature indicates that the thermal conduction performance of the GNP/PMMA nanocomposite increases, thus allowing more heat flux to be released under the same heat flux flows [38,45]. Therefore, as the coating gap and tension in the lower-layer coating increased, the thermal conduction performance of the double-layered structure’s GNP/PMMA nanocomposite improved. The difference in the heat conduction performance according to the process variables of the GNP/PMMA nanocomposite is attributable to the ink’s spreadability. Polymer nanocomposites, such as GNP/PMMA nanocomposites, differ in thermal conductivity depending on the filler content, orientation, or cohesion [46,47,48]. Among them, the lower the cohesion of the filler, the more uniform the filler distribution inside the polymer matrix, which is advantageous for the formation of a thermal conduction path in the polymer nanocomposite, and the difference in thermal conductivity of the polymer nanocomposite is up to 38% [48,49]. In this case, reagglomeration, wherein the filler aggregates during solvent evaporation, affects the degree of agglomeration of the filler [50]. In the slot-die system, the behavior of the filler inside the ink varies depending on the spreadability of the ink during solvent evaporation, and the higher the ink spreadability, the more uniform the filler [51]. Therefore, the higher the ink spreadability, the lower the cohesion of the filler, resulting in an increased thermal conductivity of the nanocomposite.

## 5. Conclusions

This study analyzed the correlation between the process variables of slot-die coating in a roll-to-roll system and the geometry of a double-layered functional layer and it was found that its upper-layer geometry can be controlled by the coating gap and tension in the lower-layer coating. In the process of manufacturing a GNP/PMMA nanocomposite in this study, changes in the surface roughness of the upper coated layer of up to 10.9% by lower-layer coating tension and up to 5.9% by the coating gap were observed. A regression analysis was conducted using the experimental planning method, confirming that tension was a dominant factor in the surface roughness of the lower and upper coated layers, more than the coating gap in the lower-layer coating. Finally, thermal conduction performance was evaluated as the TIMs of the GNP/PMMA nanocomposite to analyze the performance change in the double-layered functional layer according to process variables. Consequently, the steady-state temperature was different in each case due to the change in the ink’s spreadability according to the process variables of tension and coating gap. In the case of high ink spreadability, a lower steady-state temperature appeared, indicating excellent heat conduction performance due to the difference in filler behavior owing to the ink’s spreadability. Moreover, it was confirmed that the process variable of the slot-die coating affects the functional quality of the GNP/PMMA nanocomposite.

Based on these results, it is confirmed that the preemptive control method of the lower-layer coating process variable for controlling the geometry of the upper coated layer is applicable in the production of the functional layer of a double-layered structure. This control method does not cause crack defects in the upper coating layer, and it is expected to improve the quality and performance of the multilayered functional layer manufactured through the roll-to-roll slot-die coating system [20].

## Figures and Tables

**Table 1:** Rheological properties of the GNP/PMMA ink.

	Viscosity (Pa·s)	Solid Content (wt%)	Contact Angle (°)
Lower layer	0.00339	1.642	18.38
Upper layer	0.00378	1.038	19.43

**Table 2:** Experimental conditions.

Property	Value
Drying temperature (°C)	65
Tension (N)	18.6–55.9
Coating gap (μm)	100–300
Flow rate (mL/min)	3
Web speed (m/min)	1

**Table 3:** Experimental cases for parametric analysis.

Case	Coating Gap (μm)	Tension (N)
1	100	18.6
2		37.3
3		55.9
4	200	18.6
5		37.3
6		55.9
7	300	18.6
8		37.3
9		55.9

**Table 4:** ANOVA of the lower coated layer’s surface roughness.

Property	*p*-Value	F-Value
Coating gap	0.0005	27.75
Tension	0.0000	62.44
Coating gap × tension	0.2970	1.35

**Table 5:** ANOVA of the upper coated layer’s surface roughness.

Property	*p*-Value	F-Value
Coating gap	0.0001	56.60
Tension	0.0000	154.32
Coating gap × tension	0.0726	5.14

## Data Availability

The data presented in this study are available from the corresponding author upon reasonable request.
